# LC-MS/MS-Based Serum Metabolomics and Transcriptome Analyses for the Mechanism of Augmented Renal Clearance

**DOI:** 10.3390/ijms241310459

**Published:** 2023-06-21

**Authors:** Yidan Wang, Yifan Luo, Shu Yang, Mingyan Jiang, Yang Chu

**Affiliations:** 1 Department of Pharmacy, The First Hospital of China Medical University, Shenyang 110001, China; 2 School of Pharmacy, China Medical University, Shenyang 110122, China

**Keywords:** augmented renal clearance, metabolomics, transcriptome, vancomycin

## Abstract

Augmented Renal Clearance (ARC) refers to the increased renal clearance of circulating solute in critically ill patients. In this study, the analytical research method of transcriptomics combined with metabolomics was used to study the pathogenesis of ARC at the transcriptional and metabolic levels. In transcriptomics, 534 samples from 5 datasets in the Gene Expression Omnibus database were analyzed and 834 differential genes associated with ARC were obtained. In metabolomics, we used Ultra-Performance Liquid Chromatography-Quadrupole Time-of-Flight Mass Spectrometry to determine the non-targeted metabolites of 102 samples after matching propensity scores, and obtained 45 differential metabolites associated with ARC. The results of the combined analysis showed that purine metabolism, arginine biosynthesis, and arachidonic acid metabolism were changed in patients with ARC. We speculate that the occurrence of ARC may be related to the alteration of renal blood perfusion by LTB4R, ARG1, ALOX5, arginine and prostaglandins E2 through inflammatory response, as well as the effects of *CA4*, *PFKFB2*, *PFKFB3*, *PRKACB*, NMDAR, glutamate and cAMP on renal capillary wall permeability.

## 1. Introduction

Augmented Renal Clearance (ARC), most commonly defined as a glomerular filtration rate (GFR) of >130 mL/min/1.73 m^2^ [1], is a phenomenon of increased renal clearance of circulating solutes that has received increasing attention due to its negative effects of inadequate drug exposure, poor efficacy, and the increased risk of resistant bacteria [2,3]. ARC often occurs in critically ill people with ailments such as burns, sepsis, cancer, multiple injuries, and subarachnoid hemorrhaging [4,5,6,7]. ARC has also been reported in patients with coronavirus disease 2019 (COVID-19) [8,9,10]. The incidence of ARC in these populations can be up to 85% [1,2,11,12,13,14,15,16]. Various drugs have been reported to be affected by ARC status, such as vancomycin [17,18,19,20,21], linezolid [22], and β lactams [23,24,25,26], so the adjustment of treatment regimens for patients with ARC is also a focus of research [27,28,29,30,31,32,33].

There have been many studies on the diagnostic criteria, pharmacokinetics, and drug regimens of ARC. However, there are few reports on how ARC occurs. There are three hypotheses about the mechanisms of ARC: The theory of systemic inflammatory response syndrome, the theory of renal function reserve, and the theory of brain–kidney crosstalk [34]. The systemic inflammatory response syndrome theory suggests that inflammatory responses lead to a highly dynamic state of ARC, while targeted fluid loading therapy increases the peripheral circulatory blood flow, ultimately improving blood perfusion in the kidneys, leading to increased GFR [35,36,37]. The theory of renal function reserve suggests that nephrons are not fully dispatched under normal physiological conditions. When ARC occurs, its critical state mobilizes more nephrons, resulting in elevated GFR [38,39,40]. The theory of brain–kidney crosstalk is mainly used to explain the occurrence of ARC in brain-related diseases such as brain trauma and subarachnoid hemorrhaging. It is believed that diseases of the brain change the regulation of the central nervous system to the peripheral nerves and also alter the relevant receptors that regulate the glomerulus, resulting in the occurrence of ARC [41,42,43]. At present, these three theories lack evidence, so further research is needed.

In this study, we retrospectively collected clinical data and serum samples from ARC patients and non-ARC (NARC) patients, and analyzed the metabolites of the two groups using Ultra-Performance Liquid Chromatography Quadrupole Time-of-Flight Mass Spectrometry (UPLC-QTOF MS)-based non-targeted metabolomics methods to obtain the metabolic characteristics of the ARC patients. Transcriptomics data in the open-source Gene Expression Omnibus (GEO) database were used to screen the possible differential expression genes of ARC patients, and the metabolic pathways related to ARC were analyzed through a joint analysis of non-targeted metabolomics and the transcriptome in order to provide evidence and reasonable explanations for the pathogenesis of ARC.

## 2. Results

### 2.1. Results of Metabolomics

#### 2.1.1. Clinical Evaluation of ARC

To study the clinical features of patients with ARC, we selected relevant samples and then analyzed them. In addition, to eliminate the interference of age and body mass index (BMI) in the analysis results between the two groups, propensity score matching was performed between the ARC group and the NARC group. After the initial screening of samples, 157 samples that met the criteria were obtained, and their demographic and clinical features, and biochemical indicators are shown in Table 1. The propensity score matching tolerance was determined to be 0.16, and 102 samples were included after matching, with no significant differences in age and BMI indicators. The demographic and clinical features, and the biochemical parameters of these samples are shown in Table 2. The samples before and after matching showed significant differences in renal function indicators such as urea, creatinine, and cystatin C.

#### 2.1.2. Quality Control of Untargeted Metabolic Profiling

**Figure 1:**
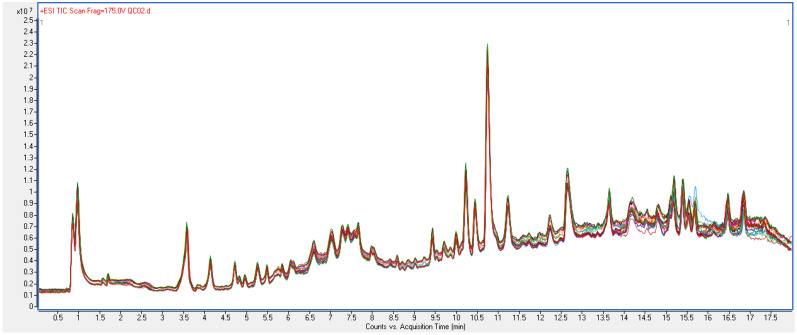
TIC pattern of quality control samples during injection sequence.

To ensure the reliability of the metabolomic data, we methodologically validated quality controller (QC) samples and examined their stability, repeatability, and replicability during data acquisition. After viewing the distribution information of the original results, a total of six parent ions were selected and named with their “mass-to-charge ratio (*m*/*z*) @ retention time (RT)”: 837.8305@0.851, 165.0791@3.634, 563.3502@5.235, 431.3025@7.675, 1485.992@10.744, and 328.1513@13.637. These six fragments were chosen because they are evenly distributed on the mass and time axes, and the peak shape is good, which can reflect the performance of the instrument at different masses and RTs. Table 3 shows the final verification results. The total ion chromatography/count (TIC) pattern expressions of the QC samples during the injection sequence were found to strongly overlap (Figure 1). These results together indicated that the instrument system offered good reproducibility stability during metabolomics analysis, and that the metabolomics data were stable and reliable.

#### 2.1.3. Screening of Differential Metabolites

**Figure 2:**
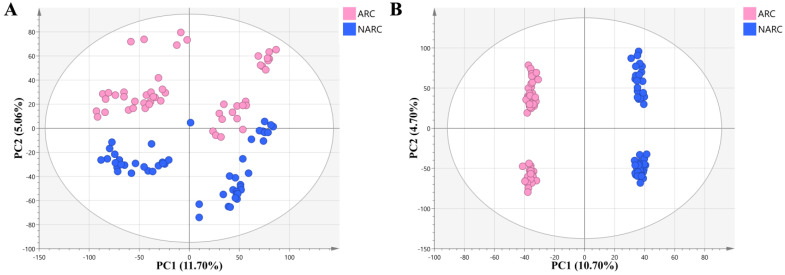
Clustering Analysis Plot: (**A**) PCA plot; (**B**) orthogonal partial least squares discriminant analysis. The color of the dots represents their grouping; the range of ellipses represents a 95% confidence interval.

**Figure 3:**
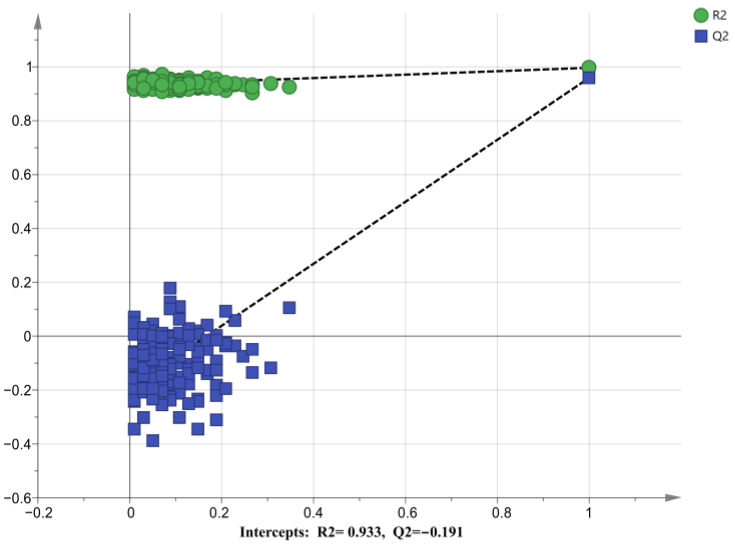
Permutation test results of OPLS-DA model. Used to validate the predictive powers of OPLS-DA models. For Q^2^, the model is considered to be not overfitted if the outcome of the result on the y-axis does not exceed 0.05. When the Q^2^ of the real model (far right) and the Q^2^ of the random label modeling are close, it indicates that the model is overfitting.

Differential metabolite screening was performed using a combination of univariate analysis, multiplier analysis, and multivariate analysis. The results of unsupervised principal component analysis (PCA) clustering (Figure 2A) showed that all samples were within a 95% confidence interval in positive ion mode. The metabolites in the ARC group and the NARC group showed a certain extent of separation tendency, and a difference in metabolic behavior was observed between the two groups. The separation trend shown in the score plot of the orthogonal partial least squares discriminant analysis (OPLS-DA) model (Figure 2B) is more pronounced, which demonstrates that the OPLS-DA model is effective in amplifying the differences between the two groups. The performance parameters of the OPLS-DA model R^2^Y were 99.7%, and those of Q^2^ were 96.0%, indicating a good fit and predictive power. The permutation test results showed that the model was not overfitted (Figure 3).

**Figure 4:**
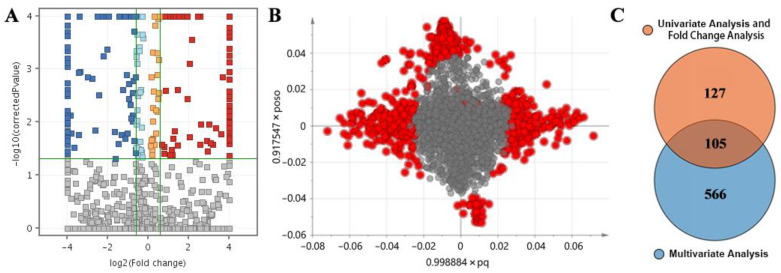
Screening results for differential metabolites: (**A**) Volcano map. The abscissa represents the results of FC analysis. The ordinate represents the univariate analysis results. Dark blue: corrected *p*-value < 0.05, FC <−1.5; Light blue: corrected *p*-value < 0.05, −1.5 < FC < 0; Orange: corrected *p*-value < 0.05, 0 < FC < 1.5; Red: corrected *p*-value < 0.05, FC > 1.5. (**B**) Score plot of the OPLS-DA model. The horizontal axis displays the x-loadings p and Y-loadings q of the predictive component. The vertical axis displays the x-loadings p(o) and the loading s(o) for the y-orthogonal component. Red indicates compounds with VIP > 1.5; (**C**) Venn plot, representing the number of differential metabolites obtained through each data analysis method.

The screening results of the differential compounds are shown in Figure 4. The combined screening results of univariate analysis and fold change (FC) analysis were visualized using volcano plots (Figure 4A), and 232 total candidate differential compounds were obtained. In total, 671 metabolites satisfied the screening conditions for multivariate analysis (Figure 4B), and 105 significantly different compounds were obtained after intersection (Figure 4C).

#### 2.1.4. Annotation Results of Differential Metabolites

**Figure 5:**
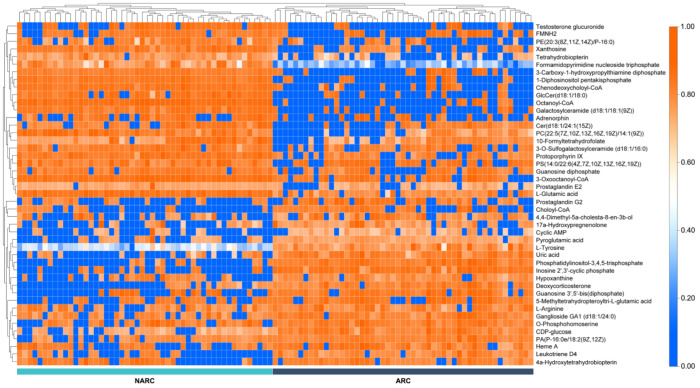
Heat map of differential metabolite expression. Each horizontal row represents a differential metabolite; each column represents a sample; the color indicates the relative expression of the differential metabolites in the individual samples. The clustering methods for horizontal rows and vertical columns are complete linkage agglomerative clustering.

In addition to 105 differential compounds screened using the above analysis methods, 45 differential compounds such as hypoxanthine, glutamic acid, and arginine were ultimately annotated. Their *m*/*z*, RT, molecular formula, HMDB ID, KEGG ID, UP/DOWN, and *p*-value are given in Table 4. “Up/Down” refers to the upregulation or downregulation of metabolites obtained via the FC analysis: “Up” means that the FC value of the metabolite is greater than 1.5 and that its content in the ARC group is upregulated relative to the NARC group; “Down” means that the FC value of the metabolite is less than −1.5, and that its content in ARC group is downregulated relative to the NARC group. “*p*-value” is the corrected *p*-value obtained via the moderated *t*-test, corrected with the Benjamini–Hochberg method. We then plotted the expression matrix to show the expression of each metabolite in each sample and its overall trend (Figure 5).

### 2.2. Results of Transcriptomics

#### 2.2.1. Included Transcriptome Datasets and Their Basic Characteristics

In transcriptomics analysis, we screened the dataset based on risk factors for the occurrence of ARC. Ultimately, five GEO datasets, GSE11374, GSE37069, GSE57065, GSE19743, and GSE28750, were included in the study. The sample information in the dataset is detailed in Table 5; 398 total samples were included in the experimental group, and 136 samples were included in the healthy control group.

**Figure 6:**
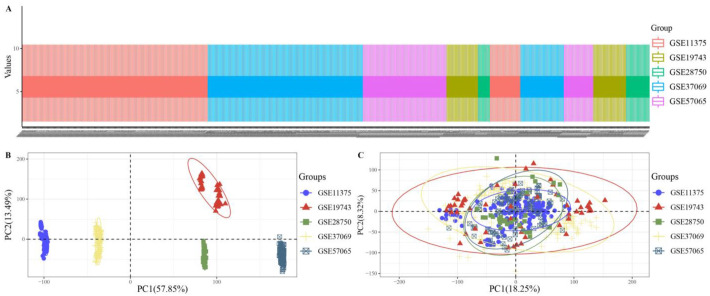
Transcriptome sample processing visualization results: (**A**) Boxplot of sample expression; (**B**) sample cluster plot before processing; (**C**) sample cluster plot after processing.

Before differential gene screening, samples were normalized and standardized to eliminate systematic errors such as batch effects. The results of sample normalization and standardized pretreatment are shown in Figure 6. In the boxplot (Figure 6A), the median and interquartile spacing levels of each sample were found to be consistent, indicating that normalization led to a consistent overall gene expression level across samples. The sample cluster plots before and after treatment (Figure 6B,C) show that normalization eliminated systematic errors between batches.

#### 2.2.2. Screening of DEGs

**Figure 7:**
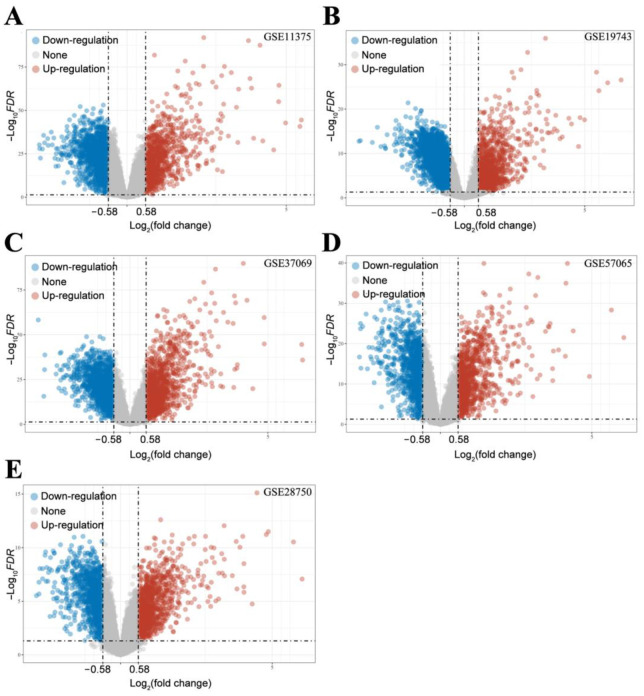
Volcano map of DEGs in each dataset: (**A**) GSE37069; (**B**) GSE57065; (**C**) GSE37069; (**D**) GSE57065; (**E**) GSE28750.

**Figure 8:**
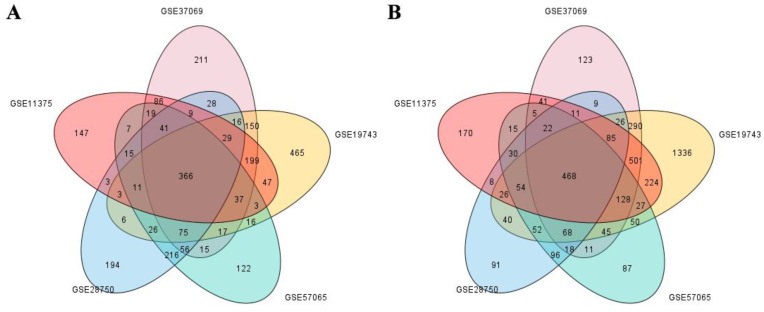
Venn diagram of different genes in each dataset: (**A**) Upregulated genes; (**B**) downregulated genes.

After processing the samples, we first performed differential genetic screening on each dataset. The screening results for the differentially expressed genes (DEGs) are shown in Figure 7. The DEGs obtained from each dataset were quantified as follows: (1) GSE11357: 1022 upregulated genes and 1815 downregulated genes; (2) GSE19743: 3420 upregulated genes and 1466 downregulated genes; (3) GSE37069: 1831 upregulated genes and 1354 downregulated genes; (4) GSE57065: 1042 upregulated genes and 1172 downregulated genes; and (5) GSE28750: 1094 upregulated genes and 1104 downregulated genes. These differential genes are thought to be associated with risk factors for ARC. In order to further target the differential genes associated with ARC, we intersected the differential genes obtained from each dataset. After taking the intersection, we finally obtained 366 upregulated genes and 468 downregulated genes related to ARC (as shown in Figure 8).

#### 2.2.3. The Results of WGCNA

**Figure 9:**
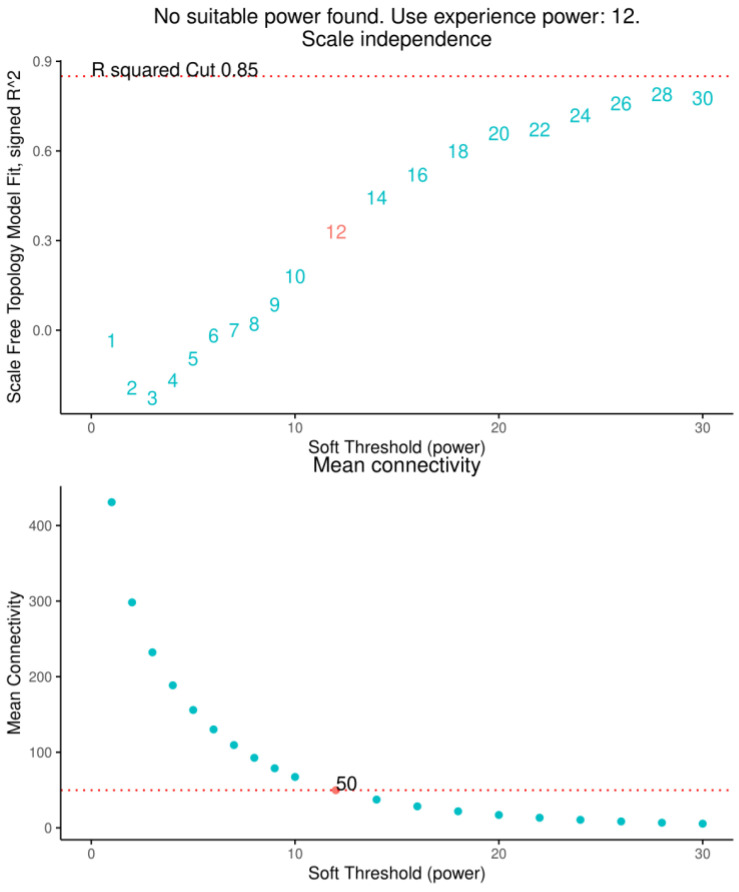
Schematic diagram of the correlation coefficient and average number of connections under different soft thresholds.

Analyzing 834 DEGs remains cumbersome, so we chose to use the weighted correlation network analysis (WGCNA) for further processing of the DEGs. In total, 14 outlier samples were removed according to the Z-score value, and 520 samples were retained for the construction of the subsequent weighted gene co-expression network. The network topology analysis plot (Figure 9) determined that the optimal soft threshold β should be 12, under which the comprehensive performance of the correlation coefficient and average number of connections were good. The average number of connections between genes was 50.

**Figure 10:**
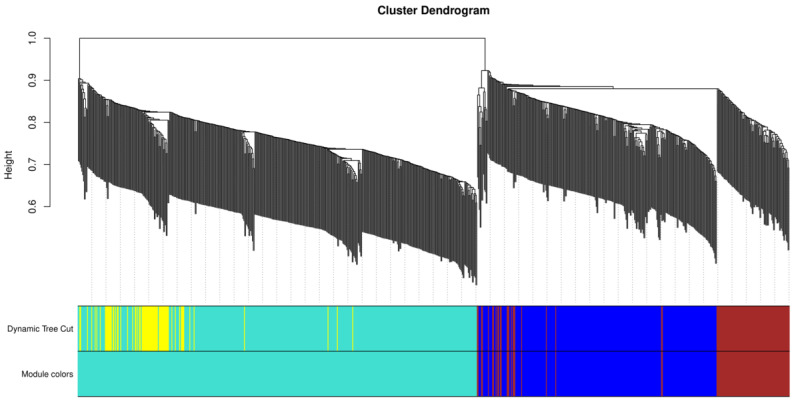
Cluster dendrogram of DEGs. Different branches of the cluster tree represent different genes, and different colors represent different modules. Genes in the same color module indicate similar expression patterns.

**Figure 11:**
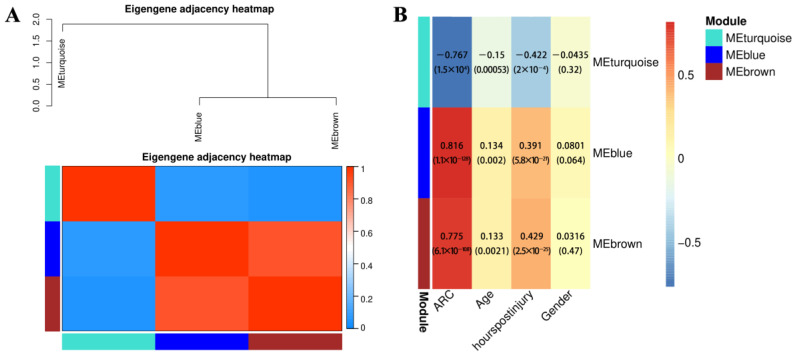
Correlation heatmap: (**A**) Module-to-module dependencies; (**B**) correlation between the module and sample characteristics.

**Figure 12:**
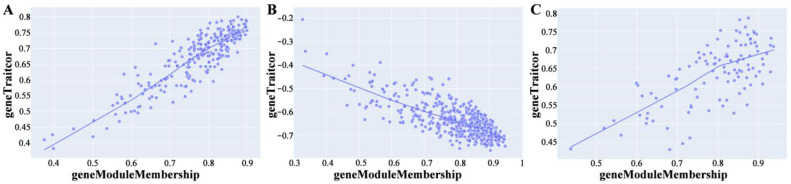
Gene–trait scatterplot: (**A**) Blue; (**B**) turquoise; (**C**) brown. Each dot represents a DEG, and the line is the correlation fitting curve of the module with the ARC trait.

The dynamic cut tree ultimately divided the 834 DEGs into 3 modules (Figure 10); the blue module contained 257 DEGs, the turquoise module contained 468 DEGs, and the brown module contained 109 DEGs. The correlation heat map showed a strong correlation between the three modules, with a certain interaction in function (Figure 11A). The correlation coefficients between the blue, green, and brown modules and ARC were 0.816, −0.767, and 0.775, respectively (Figure 11B), with the best correlation being found between the blue module and ARC. The three module-ARC scatterplots (Figure 12) depict the correlation between genes and ARC in different modules. Here, the genes in the blue module and the brown module have a good linear positive correlation with ARC, while those in the turquoise module have a good linear positive correlation with ARC. The correlation curve in the subplot has a large slope and is linear, indicating linear correlation with the occurrence of ARC. Gene scattering was evenly scattered near the module curve, indicating that module eigengene (ME) could better represent the expression patterns of genes in the module when analyzing the correlation between the module and ARC.

**Figure 13:**
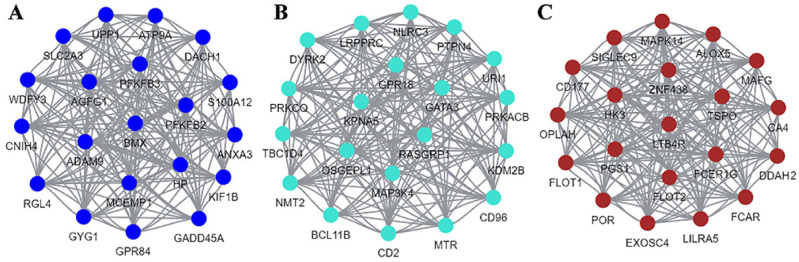
Hub genes visualization network diagram: (**A**) Blue; (**B**) turquoise; (**C**) brown.

Lastly, the first 20 genes were obtained in each module as the hub genes of the corresponding modules, and a visual network (see Figure 13) was drawn to represent the interactions between the hub genes.

### 2.3. Results of Pathway Analysis

#### 2.3.1. The Result of Network Analysis

**Figure 14:**
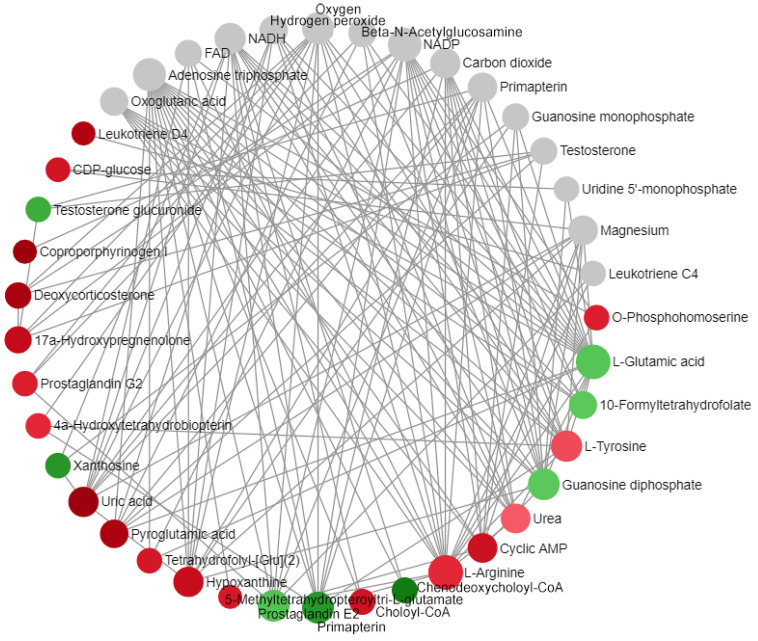
Metabolite-metabolite network diagram: A red dot indicates upregulation, a green dot indicates downregulation, and a gray dot indicates that this metabolite is not included in the metabolite that we ultimately annotated (Table 4), but is closely related to other differential metabolites. The size of the dot represents the magnitude of the FC.

We performed a metabolite-metabolite network analysis to annotate the interaction relationship between metabolites. Ultimately, a metabolic network with node number 41, connection number 145, and seed number 26 was obtained (as shown in Figure 14). The seed information in the network is shown in Table 6.

**Figure 15:**
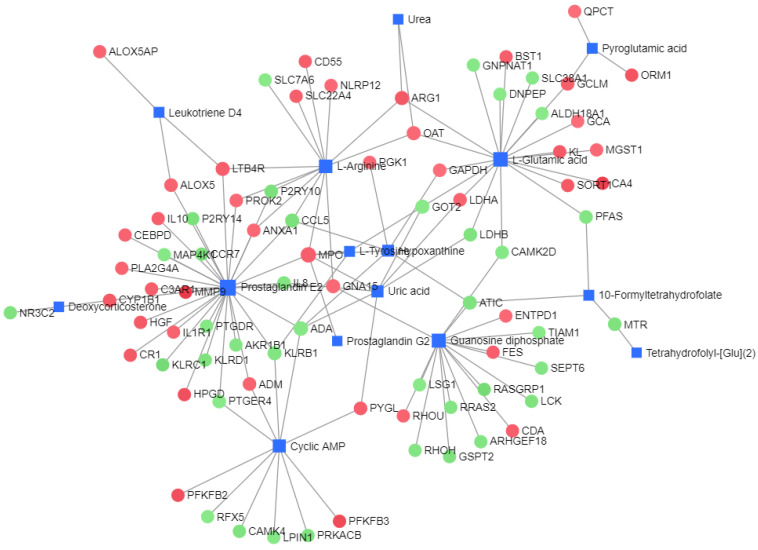
Gene-metabolite network diagram: A square represents metabolites, and round shapes represent genes. Red indicates upregulation, and green indicates downregulation.

The gene-metabolite network analysis yielded a gene metabolite network with a node number of 95, an edge number of 120, and a seed number of 95, as shown in Figure 15). In total, 19 genes were found to be involved in L-glutamate regulation: *PFAS*, *SORT1*, *CA4*, *GCA*, *GOT2*, *LDHA*, *ARG1*, *OAT*, *BST1*, *CAMK2D*, *GAPDH*, *KL*, *LDHB*, *DNPEP*, *MGST1*, *ALDH18A1*, *SLC38A1*, *GCLM*, and *GNPNAT1*. There were 13 genes involved in L-arginine regulation: *ARG1*, *OAT*, *MPO*, *GNA15*, *ANXA1*, *CCL5*, *LTB4R*, *CD55*, *PROK2*, *SLC22A4*, *P2RY10*, *NLRP12*, and *SLC7A6*. There were 11 genes involved in cAMP regulation: *KLRB1*, *LPIN1*, *CAMK4*, *PTGER4*, *PRKACB*, *PFKFB3*, *ADA*, *ADM*, *RFX5*, *PYGL* and *PFKFB2*. There were 30 genes involved in the regulation of prostaglandin E2: *MPO, KLRB1*, *GNA15*, *PTGER4*, *ADA*, *ADM*, *ANXA1*, *CCL5*, *LTB4R*, *PROK2*, *P2RY10*, *HGF*, *MMP9*, *CCR7*, *CYP1B1*, *ALOX5*, *IL8*, *PLA2G4A*, *CEBPD*, *IL10*, *C3AR1*, *PTGDR*, *P2RY14*, *MAP4K1*, *IL1R1*, *HPGD*, *CR1*, *AKR1B1*, *KLRD1*, and *KLRC1*.

#### 2.3.2. Pathway Analysis

**Figure 16:**
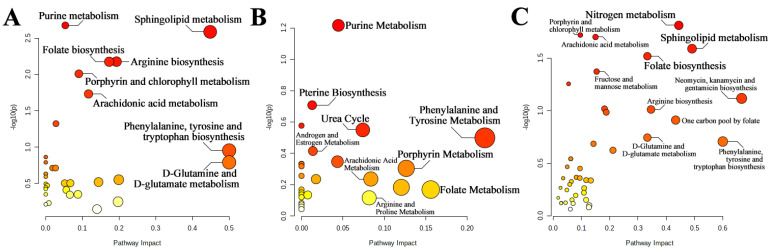
Pathway analysis enrichment bubble diagram: (**A**) Only differential metabolites were used for the results of the pathway analysis in KEGG; (**B**) Only differential metabolites were used for the results of pathway analysis in the SMPDB; (**C**) Joint-pathway analysis results of differential genes and differential metabolites in combination. The size of the bubbles is determined by the pathway impact values from the pathway topology analysis, and the larger the value, the larger the bubble. The color of the bubbles is determined by the *p*-values from the pathway enrichment analysis. The larger the *p* -value, the darker the color (reddish), while the smaller the *p*-value, the lighter the color (yellow or even white).

The results of the pathway analysis of differential metabolites using the Kyoto Encyclopedia of Genes and Genomes (KEGG) database are shown in Figure 16A, including purine metabolism, arginine biosynthesis, sphingolipid metabolism, folate biosynthesis, and arachidonic acid metabolism. The pathway analysis of differential metabolites using The Small Molecule Pathway Database (SMPDB) database is shown in Figure 16B, and the enrichment results feature a total of 26 metabolic pathways. The results of the joint-pathway analysis are shown in Table 7 and Figure 16C.

#### 2.3.3. Mechanism Analysis

**Figure 17:**
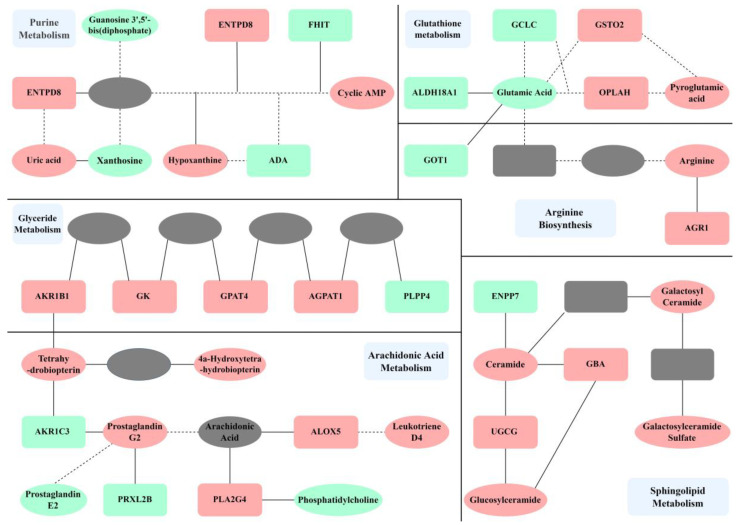
Schematic diagram of changes in the pathways of ARC patients. Solid lines represent direct interactions, and dashed lines represent indirect interactions. Red indicates metabolites or genes upregulated, green indicates metabolites or genes downregulated, and gray indicates omitted metabolites or genes that were not included in our study results.

**Figure 18:**
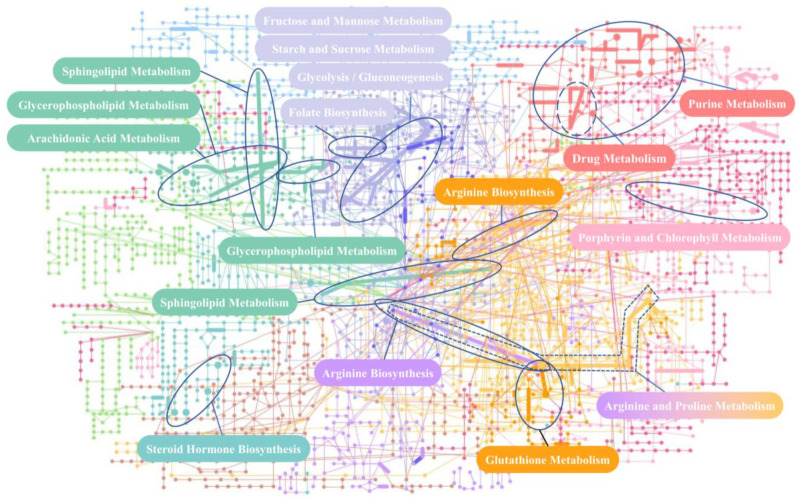
Global metabolic network changes in ARC patients. Circles and bold lines indicate changes in metabolic pathways that may occur in patients with ARC.

**Figure 19:**
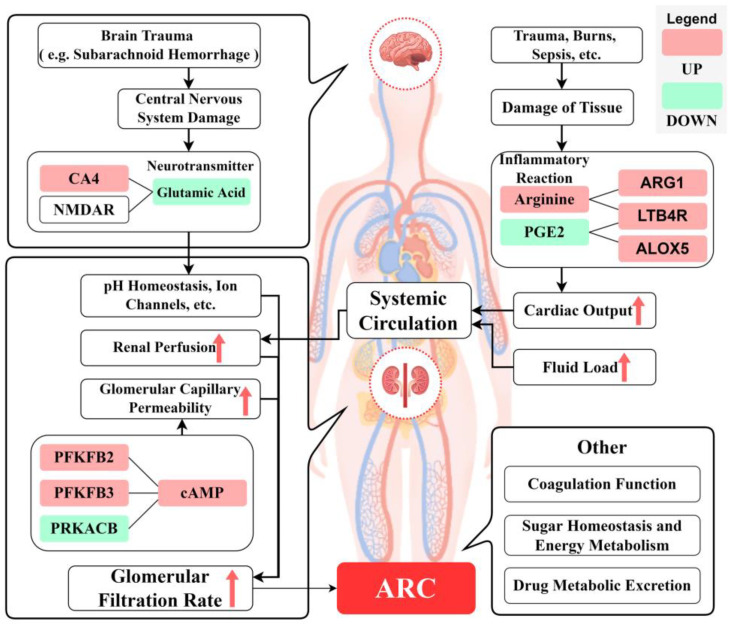
ARC pathogenesis diagram.

We plot the enrichment results of the ARC pathway, as well as significant changes in differential metabolites and DEG nodes in Figure 17, to demonstrate which differential metabolites and genes are included in the pathway and their interactions. We draw the overall metabolic spectrum changes of ARC in Figure 18 to demonstrate the relationships between various metabolic pathways. Finally, the pathogenesis of ARC was speculated and plotted in Figure 19. The detailed inference process can be found in the Discussion section.

## 3. Discussion

Genes exist upstream of metabolic regulation, and after the transcription stage, subsequent translation, post-translational modification, environmental factors, etc., will affect the final metabolic outcome. Therefore, in this study, we chose to start with the differential metabolites of ARC. First, we sought to determine the roles of differential metabolites in the occurrence of ARC, and then we explored the regulatory role of DEGs in ARC. It can be seen from the seeds with a large number of nodes in the metabolite-metabolite network analysis results (Figure 14 and Table 6) and the gene-metabolite network analysis results (Figure 15) that L-glutamic acid, L-arginine, cAMP, and prostaglandin E2 were associated with the occurrence of ARC. In addition, the effects of upregulated *CA4* on L-glutamate, upregulated *LTB4R* on L-arginine, downregulated *PRKACB*, upregulated *PFKFB3*, and *PFKFB2* on *cAMP*, and upregulated *LTB4R* and *ALOX5* on prostaglandin E2 may represent common changes in ARC patients. These differential metabolites and pivotal genes are of high value in studying the pathogenesis of ARC. We attributed the effects of these differential metabolites and differential genes on ARC to improving renal perfusion and improving glomerular capillary wall permeability, and summarized them in Figure 17.

### 3.1. Improving Renal Perfusion

The change of renal blood perfusion in the ARC state is mainly realized through the regulation of the inflammatory response by L-arginine, L-glutamic acid, and prostaglandin E2.

L-Arginine is a substrate for the production of nitric oxide (NO) via nitric oxide synthase (NOS). NO is a key signaling molecule in cardiovascular physiology and pathology, with negative chronotropic, negative, or positive inotropic effects, as well as positive gonadotropin effects [44,45]. NOS is a double-plus oxidase consisting of reductase and oxidase domains. NOS and O_2_ oxidize guanidine nitrogen from L-arginine to produce NO and L-citrulline [46,47,48]. This reaction requires the involvement of flavin mononucleotide (FMN), flavin adenine dinucleotide (FAD), and tetrahydrobiopterin. The depletion of L-arginine and/or tetrahydrobiopterin leads to the uncoupling of NOS, resulting in O_2_ substituting L-arginine as an end electron acceptor and leading to the formation of superoxide [49]. The further binding of superoxide to NO leads to the production of peroxynitrite [50], which can lead to the development of congestive heart failure through cell damage and reduced myocardial contractility [51]. The production of peroxynitrite by NOS exposed to low L-arginine concentrations in renal cell lines has also been reported [52]. In addition, studies have shown that patients with sepsis exist in a state of impaired arginine, and mice with sepsis were observed to experience a decrease in arginine within 90 min of lipopolysaccharide (LPS) infusion [53]. Based on the above evidence, patients suffering from sepsis with impaired arginine may be at risk of renal hypoperfusion, resulting in a decreased GFR due to decreased myocardial contractility and decreased cardiac output, resulting in the obstruction of peripheral circulation.

In this study, the upregulation of arginase *ARG1* in transcriptomic results was consistent with the increased arginine consumption observed in patients with sepsis. However, in the metabolomic results, L-arginine in the ARC group showed upregulation, suggesting the presence of other unknown pathways that increased the source of arginine to counter the increased arginine catabolism caused by ARG1 upregulation. Upregulated L-arginine provides sufficient conditions for the production of NO, which can reduce cell damage and improve cardiac output, ultimately increasing renal blood flow and the GFR. Arginine and tetrahydrobiopterin supplementation in rats with salt-induced elevated blood pressure was previously shown to improve renal hemodynamics with increased renal perfusion [54], consistent with the results of this study.

Glutamate was downregulated in the experimental group. It is naturally produced in the body and performs a variety of functions in the body, with circulating glutamate being filtered in the glomerulus and reabsorbed in the proximal tubule [55]. In the fasted state, the kidneys metabolize glutamine absorbed from the blood to produce ammonia and glutamate [56]. The glutamate-regulated N-methyl-D-aspartate receptor (NMDAR) is a glutamate-gated nonselective cation channel that is highly expressed in the kidneys, in addition to being widely expressed in the central nervous system [57,58]. Multiple reports have suggested that NMDAR plays an important role in maintaining renal homeostasis [58,59], and that its overexpression or inhibition may cause renal homeostasis imbalance and lead to the occurrence of renal disease. Studies have shown that the regulation of NMDAR agonists can change the GFR and the GFR of mononephron units [60,61], so it is speculated that the change of glutamate on NMDAR regulation may play an important role in ARC genesis.

The interaction between L-glutamate and L-arginine is of particular interest. This interaction indirectly improves renal blood perfusion through L-arginine. In addition, NO has a positive gonadotropin effect, and the enrichment results for the metabolic pathway in the SMPDB database also showed changes in androgen and estrogen metabolism, which may be the reason for why ARC exhibited more of this epidemiological feature in male patients.

Prostaglandin E2 is produced by all kidney cells and is the most abundant prostaglandin that plays an important physiological and pathological role in the kidneys. For example, this prostaglandin is involved in regulating the reabsorption of osmotic water through vasopressin, regulating water metabolism in the body [62], and improving renal function through a variety of antioxidant, anti-apoptotic, and inflammatory inhibitory effects. In the inflammatory response, prostaglandins, lipid signaling molecules involved in pain and inflammation, enhance tissue regeneration processes after injury in different organ systems, and regulate hematopoietic stem cell/progenitor cell homeostasis to regulate hematopoietic function [63]. LTB4R, ALOX5, and prostaglandin E2 are involved in the arachidonic acid metabolism pathway. The potent chemoattractant and proinflammatory mediator LTB4 is synthesized from arachidonic acid by ALOX5 [64].Multiple risk factors for ARC such as trauma [64], sepsis [65], cancer [66], etc., also found differences in LTB4R expression. Additionally, studies have found that ALOX5 can improve kidney function by weakening the inflammatory response [67], which is also consistent with the upregulation of ALOX5 in the ARC population in this study. In this study, prostaglandin E2 was found to be downregulated, which reduced the inflammatory inhibition effects on ARC patients and also regulated tubular water and sodium transport, glomerular filtration, and vascular resistance through receptors, ultimately leading to the occurrence of ARC.

### 3.2. Improving Glomerular Capillary Wall Permeability

The glomerular capillary wall is a dynamic system that forms the glomerular filtration barrier. The permeability of this wall depends on three layers: endothelial cells, basement membranes, and podocytes [68]. Podocytes are terminally differentiated and highly specialized cells covering the outer surfaces of glomerular capillaries, and the dynamic structures formed by their foot processes and fissure septa determine the final size of the glomerular filtration barrier, which is essential for maintaining the integrity of the glomerular filtration barrier.

Purinergic signaling is involved in a variety of physiological processes in the renal system, including the regulation of glomerular filtration. cAMP is a universal second messenger that has multiple effects on kidney function, in addition to regulating vasoactivity and regulating downstream protein targets (including ion channels, enzymes, and transcription factors) through cAMP-dependent protein kinase A (PKA). The findings suggest that the cAMP-PKA signaling pathway in podocytes may regulate actin cytoskeletal organization for glomerular protection [69], as well as the GFR [69]. At the same time, cAMP levels were found to be reduced in fibrotic kidney tissue, and cAMP supplementation improved tubular atrophy and extracellular matrix deposition [70].

In this study, the metabolomics results showed that cAMP in the purine metabolic pathway exhibited upregulation in the ARC group, with upregulated cAMP enhancing the size of the glomerular filtration barrier and reducing extracellular matrix precipitation, which ultimately manifested as an increase in the GFR. The upregulation of cAMP may be the result of a combination of upregulating the pivot genes *PRKFB2* and *PRKFB3*, and downregulating *PRKACB*, among which *PRKFB2* and *PRKFB3* are involved in the AMPK signaling pathway.

AMPK is an important cellular energy sensor that is activated in response to intracellular ATP depletion and plays a role in restoring energy homeostasis by activating ATP production and inhibiting ATP consumption pathways [69]. In addition, carbohydrate metabolism is an important source of energy production. In our transcriptomics analyses, we found changes to the insulin signaling pathways in ARC. Moreover, previous studies have shown that glucagon increases the concentration of cAMP in the blood [71], suggesting that the regulatory effect of cAMP on the GFR may be related to carbohydrate homeostasis and energy stability. Glucagon increases the concentration of cAMP in the blood, affecting proximal tubular reabsorption. The authors in [71] showed that low-dose glucagon increases GFR only when combined with an infusion of cAMP (mimicking glucagon-induced hepatic release). The above evidence suggests that patients with critical illnesses may exist in an imbalanced state of energy metabolism and carbohydrate metabolism. The resulting changes in the insulin signaling pathway and AMPK signaling pathway work together to upregulate cAMP, and upregulated cAMP causes an increase in the GFR in the ARC state by changing the permeability of the glomerular capillary wall.

### 3.3. Limitations of This Study

The results for the ARC and NARC plasma metabolomesin in this study indicated that there are indeed differences in metabolic patterns between the ARC and NARC populations, providing meaningful information for exploring the pathogenesis of ARC. However, this study also has the following limitations to be considered. (1) The serum metabolomics samples in this study were collected retrospectively, while transcriptomics uses a public database. Thus, the correlation between the two was insufficient. Therefore, a prospective study on the collection of serum samples and transcriptome samples should be carried out to obtain more comprehensive validation. (2) The method used in this study was semi-quantitative non-targeted metabolomics analysis. In addition, our metabolite annotation was mainly based on consistency with the *m*/*z* and MS/MS fragments in the database. To validate the above results, a targeted approach with authentic standards should be used in a future validation study. (3) During the metabolomics analysis of data, the results of OPLS-DA and PCA showed a split within the same phenotypic group. We only excluded the possibility of categorical variables such as gender, department, and clinical outcome, but we have not yet found the specific reasons for this phenomenon. (4) This study found the potential mechanism of ARC, but no in-depth study was carried out. Future studies should be performed in the cell line or by using animal models.

## 4. Materials and Methods

### 4.1. Methods for Metabolomics

#### 4.1.1. Information Collection and Sample Screening

The present study used a retrospective collection of vancomycin blood concentration monitoring samples from patients suspected of or diagnosed with Gram-positive bacterial infections in the First Affiliated Hospital of China Medical University, from 1 January 2017 to 31 October 2020. According to the creatinine clearance rate (CrCL) and vancomycin blood trough concentration results (C_trough_), the inclusion criteria of the study were established as follows: (1) experimental group (ARC group): CrCL > 130 mL/min and Ctrough < 10 mg/L, and (2) control group (NARC group): 80 mL/min ≤ CrCL ≤ 130 mL/min and 10 mg/L ≤ Ctrough ≤ 20 mg/L. Exclusion criteria were as follows: (1) age < 18 years or age > 80 years; (2) pregnant or lactating women; (3) missing samples; (4) non-valley concentration of blood sample; (5) serum sample collection time < 48 h after first administration. We completed the preliminary screening of samples according to the above criteria. CrCL was calculated using the Cockcroft-Gault formula [72]. SPSS v22.0 software was used to match the propensity scores of the primary screening samples, with age and BMIas the predictive variables. Finally, two groups of samples were obtained for subsequent statistical and metabolomics analyses.

#### 4.1.2. Sample Preparation

Serum samples stored in an ultra-low temperature freezer at −80 °C were taken and dissolved in a 4 °C freezer. Next, 30 μL of each serum sample was placed in a 1.5 mL centrifuge tube, and 150 μL of pre-refrigerated methanol was added. The solution was then vortexed for 2 min to achieve the purpose of mixing homogeneous and precipitating proteins. The mixture was centrifuged at 13,000 rpm (centrifugal force = 15,115× *g*) for 10 min at 4 °C, and then the clear upper liquid was aspirated for detection.

#### 4.1.3. Quality Control

In total, 20 μL of each sample was mixed to prepare (QC) samples. The reliability of the metabolomic data was confirmed primarily using these samples. Before the formal analysis of the sample, the method was verified by calculating the RSD indicators of the corresponding peak area and RT of the reference ions. (1) System repeatability: The same QC sample was injected five times in a row to evaluate the suitability of the instrument system. (2) Method replicability: Five QC samples were processed in parallel to evaluate the applicability of the sample treatment method. (3) Sample stability: The dissolved serum sample was placed at room temperature to investigate the stability of the sample after 4 h, 8 h, and 12 h. Deviations throughout the injection process were evaluated by interspersing one QC sample every 10 samples. The RSD of the peak area of the corresponding sample was less than 5.0%, and the RSD of its RT was less than 1.5, indicating good data quality.

#### 4.1.4. LC–MS Analysis

UPLC-QTOF MS analyses of the samples were conducted using an Agilent Q-TOF LC/MS 6530 system. A UPLC HSS T3 column (1.8 μm, 2.1 mm × 100 mm) (Waters, Milford, MA, USA) was used for reverse-phase separation. Plasma metabolites were separated with a 20 min gradient at a flow rate of 0.3 mL/min. Mobile phase A was 0.1% formic acid in H_2_O, and mobile phase B was 0.1% formic acid in acetonitrile. The gradient was set as follows: 0–1 min, 1% solvent B; 1–8 min, 1–65% solvent B; 8–18 min, 65–99% solvent B; and 18–20 min, 1% solvent B. The column temperature was set as 40 °C. The mass spectrometer was operated in positive ion mode within the range of 100–1700 *m*/*z*. The acquired data were used in the screening process of the metabolites.

After screening out differential metabolites, we further annotated these metabolites by collecting data from the QC samples in targeted MS/MS mode. In this mode, the daughter ion fragments were obtained through the targeted collision of specific *m*/*z* parent ions in a certain time range. The collision energy was optimized as 10 v, 20 v, and 40 v for each target. The fragmentation information of the metabolites obtained above at different collision energies was used to annotate the metabolites.

#### 4.1.5. Data Processing

**Figure 20:**
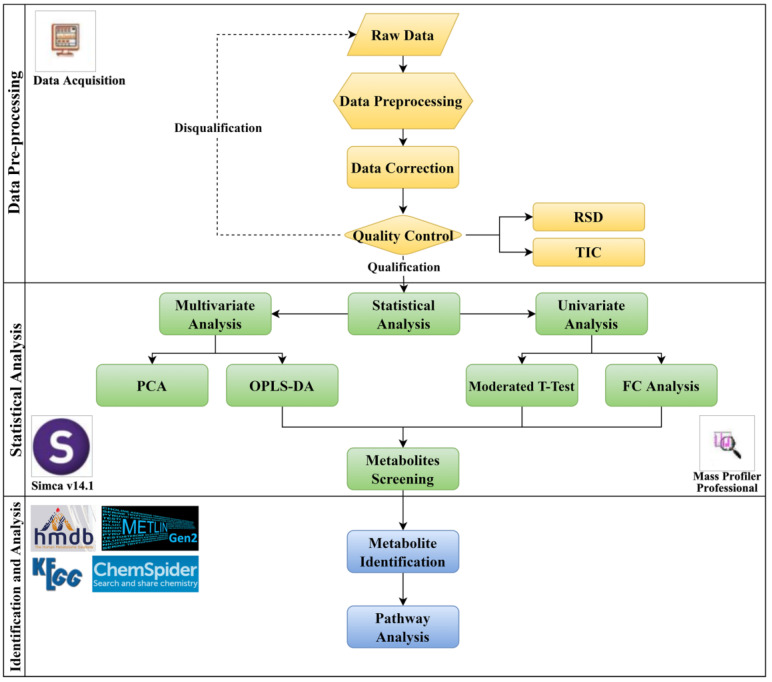
Differential metabolite analysis process.

The entire data analysis process is shown in Figure 20. We used a Mass Hunter Profinder (B.08.00, Agilent, Technologies, Inc., Santa Clara, CA, USA) and Mass Profiler Professional (MPP, v14.9.1, Agilent, Technologies, Inc., Santa Clara, CA, USA) to preprocess the raw data. The specific steps are: (1) Molecular feature extraction: Molecular information is extracted from the original data features, and the molecular characteristics are initially integrated through the processes of noise filtering, classification and alignment, and post-processing filtering; (2) Recursive processing of compound mass spectrometry information: Based on the ion information in the original data, the ion peak of the compound is attributed, peak integration and filtering are performed, the extraction spectrum is extracted, and post-processing and filtering are carried out again, based on the accurate mass and mass spectrometry characteristic data. After the above steps, each sample is given a list of compounds with molecular weight, RT, *m*/*z*, and abundance, and is displayed in “.CEF” format output. Then, the experimental group and the control group samples of the corresponding “.CEF” file are imported into MPP software for normalization and standardization, and a “metabolite-abundance” data matrix is obtained for differential compound screening.

The univariate analysis method was a moderated *t*-test corrected with the Benjamini-Hochberg method. Multivariate analysis used SIMCA (v17.0.2, Biotree Biomedical Technology Co., Shanghai, China). Unsupervised PCA was used to describe within-group similarities and between-group differences. OPLS-DA was used as a supervised principal component analysis method to zoom in on differences and to further screen for differential metabolites. The final screened differential metabolites met all three conditions at the same time: (1) a False Discovery Rate (FDR) in the univariate analysis of <0.05; (2) FC analysis results of |log_2_(FC)| ≥ 0.58; and (3) a corresponding variable importance in the projection (VIP) of >1.5 in the OPLS-DA model.

#### 4.1.6. Metabolite Annotation

According to the MS information for the metabolites (monoisotopic peaks and isotope peaks of different adducts) and MSMS information (characteristic fragment peaks after fragmentation of the parent ions), an annotation of the differential metabolites was completed through comparison with the Human Metabolome Database (HMDB) (http://www.hmdb.ca/), KEGG (http://www.kegg.com/), ChemSpider (http://www.chemspider.com/), Metlin (http://metlin.scripps.edu/), and MassBank (https://massbank.eu/). We visited these websites between 1 November 2021 and 15 January 2022. Successfully annotated metabolites satisfy the condition that the matching tolerance of the parent ion is less than 50 ppm, while the *m*/*z* and relative response of more than 2 fragment ions are consistent with the database.

### 4.2. Transcriptomics Method

#### 4.2.1. Dataset Filtering and Preparation

In the GEO database (https://www.ncbi.nlm.nih.gov/geo/, accessed between 4 July 2021 and 23 August 2021) of the National Biotechnology Center of the United States, ‘Burn’, ‘Sepsis’, and ‘Trauma’ were used as search terms, and the species was limited to ‘Homo sapiens’. The inclusion criteria were as follows: (1) the sample type was a human serum sample; (2) the sample groups included a disease group and a healthy control group; (3) the study included the determination of the messenger ribonucleic acid (mRNA) expression profile; and (4) the disease duration was ≤120 h. Exclusion criteria were as follows: (1) age > 80 years and age < 18 years. (2) pregnant or lactating women.

We downloaded the raw gene expression data as MINIML files from the GEO database. The probe treatment scheme was as follows: (1) remove probes without the corresponding genes; (2) when multiple probes corresponded to the same gene, the average value of all corresponding probes was selected. The data preprocessing process was as follows: (1) for sample normalization, Log2 transform was used; (2) sample standardization was used for the R v3.4.1 platform (the normalize.quantiles function in the preprocessCore package from http://www.r-project.org/, accessed on 8 September 2021); (3) we removed the batch effect via the remove BatchEffect function in the limma package of the R software; (4) for probe annotation, according to the platform annotation file, the probe ID was converted into the gene name. After the above probe processing and data preprocessing, the corresponding gene expression matrix was obtained.

#### 4.2.2. DEGs Screening and Annotation

DEGs were screened using the limma package in R software, including the construction of a standard data matrix, the creation of a comparative model, linear fitting, difference calculation, and statistical testing. The final screening was based on the significance of the statistical test and the FC. The methods and parameters were as follows. (1) Univariate analysis: Student’s *t*-test was used to calculate the *p*-value of the gene expression difference between the disease group and the healthy control group in each dataset, and the *p*-value of the *t*-test was corrected using the Benjamini-Hochberg method. The screening condition was FDR < 0.05. (2) For differential expression fold screening, the threshold used was |log_2_(FC)| ≥ 0.58. After obtaining the DEGs of each dataset through the above threshold, intersection processing was performed, and the obtained intersecting DEGs were identified as genes that are related to ARC.

#### 4.2.3. WGCNA Analysis

The WGCNA of the DEGs obtained in Section 2.2.2. was performed using the WGCNA package in the R software. The specific steps included the following steps. (1) Data preparation, which involved preparing the DEG expression profiles and clinical feature information table for the specimens. (2) Eliminating outliers, which used −2.5 as the Z-score cutoff value to eliminate outliers. (3) Construction of the gene correlation matrix: The correlation between genes was calculated using the Bicor function. (4) Converting to the adjacency matrix and constructing the weighted gene co-expression network: The key parameter in this process was determined as the soft threshold β with the network topology analysis diagram, and the maximum traversal value of the soft threshold was 30. (5) Dynamic tree cutting to generate the co-expression modules: We set the tree branch depth to 2, the minimum module gene size to 25, and merged related modules. (6) Calculating the correlation between module–module and module–clinical features: Calculation of the module was completed with ME using Pearson correlation analysis. Through the above steps, we identified the association between the module and the occurrence of ARC, and selected the first 20 genes with the largest weight values in the relevant module as the hub genes for ARC occurrence.

#### 4.2.4. Gene Function Enrichment Analysis

ID conversion used the org.Hs.eg.db package (v3.10.0) and enrichment analysis used the clusterProfiler package (v3.14.3) in the R software [73]. The Gene Ontology (GO) and the KEGG databases were selected to enrich the hub genes. (1) GO was used to enrich cellular component (CC), molecular function (MF), and biological process (BP) hub genes. (3) The KEGG PATHWAY library and the KEGG ORTHOLOGY library were used to enrich the metabolic pathways.

### 4.3. Method of Pathway Analysis

The action network was drawn to understand the relationship between metabolites–metabolites and gene-metabolites in the DEGs of transcriptomics and differential compounds of metabolomics. Using the network analysis function of the online analysis tool Metaboanalyst 5.0 (https://www.metaboanalyst.ca/, accessed between 20 January 2022 and 27 January 2022), the input node information and corresponding change multiples were mapped to an interactive network, and the network algorithm was based on the desparse graphical lasso modeling process [74,75]. The association between nodes was extracted from STITCH, and the final output contained a subnet of at least three nodes, with only the shortest path being retained between the various sub-networks. The network results included (1) the gene-metabolite interaction network describing the interactions between genes and metabolism, and (2) the metabolite-metabolite interaction network describing the interactions between metabolites.

Pathway changes for DEGs and differential metabolites were analyzed using the joint-pathway analysis function in the online analysis tool Metaboanalyst 5.0. Analysis. The visualization method was a scatter diagram, the enrichment method was a hypergeometric test, the topological measurement method was degree centrality, the integration method was combinatorial query, and the selected pathway database was the metabolic pathway (synthesis), which excluded regulatory pathways containing only genes.

The obtained information was integrated to analyze the mechanisms of ARC. The main nodes of the changes in each pathway were extracted, and the changed metabolic pathways were drawn in the KEGG global metabolic network (KO01100) to holistically visualize the role of each pathway in the occurrence of ARC and to draw a mechanism map of ARC occurrences.

## 5. Conclusions

(1) ARC patients exhibit the upregulation of arginine under the combined effects of *LTB4R* and *ARG1*. Although the upregulation of *ARG1* indicates an increase in arginine consumption in critically ill patients, *LTB4R* may have a more positive improvement effect, ultimately improving renal blood flow perfusion and leading to the occurrence of ARC through NO-related inflammatory reactions. (2) Glutamic acid not only directly regulated the GFR through NMDAR, but also indirectly improved renal blood perfusion through the glutamine pathway and arginine synthesis pathway, resulting in the occurrence of ARC. (3) Under the joint regulation of *LTB4R* and *ALOX5*, downregulated prostaglandin E2 led to ARC by indirectly regulating inflammatory pathways and directly adjusting tubular water and sodium transport, glomerular filtration, and vascular resistance. (4) *PRKFB2*, *PRKFB3*, and *PRKACB* affected cAMP through the AMPK signaling pathway and insulin signaling pathway, and changed the permeability of the glomerular capillary wall and extra stromal precipitation, resulting in ARC.

## Figures and Tables

**Table 1:** ARC and NARC clinical data before propensity score matching.

Items	ARC (N = 76)	NARC (N = 81)	*p*-Value
Gender (Male/Female)	42/34	53/28	0.193
Age (years)	62 (55.25–66.75)	50 (38–59)	0.000 *
Vancomycin concentration (mg/L)	14.1 (12.15–16.68)	7.1 (5.25–8.2)	0.000 *
BMI (kg/m^2^)	23.68 (21.48–26.28)	25.66 (23.13–27.89)	0.001 *
Creatinine clearance (mL/min)	101.55 (92.21–115.59)	171.45 (148.38–208.7)	0.000 *
White blood cells (10^9^/L)	9.05 (5.93–13.23)	8.98 (5.51–12.68)	0.562
Lymphocyte count (10^9^/L)	1.13 (0.84–1.49)	1.25 (0.78–1.73)	0.497
Neutrophil count (10^9^/L)	6.55 (3.96–11)	6.66 (3.39–9.61)	0.256
Monocytes count (10^9^/L)	0.54 (0.4–0.76)	0.57 (0.35–0.81)	0.868
Proportion of neutrophils (%)	76.5 (69–86.7)	73.4 (63.7–81.35)	0.074
Red blood cells (10^12^/L)	3.26 ± 0.71	3.34 ± 0.82	0.517
Hemoglobin (g/L)	97 (82–110)	98 (79.5–118)	0.685
Platelet count (10^9^/L)	204 (116–300)	252 (133–346.5)	0.198
Aspartate aminotransferase (U/L)	20 (15.5–35)	23 (14–49.5)	0.299
Alpine aminotransferase (U/L)	27 (17–49)	33 (20.5–62.5)	0.156
Alkaline phosphatase (U/L)	90 (69–125)	87 (61.5–132)	0.566
Glutamyl transpeptidase (U/L)	47 (24–100)	65 (31–102)	0.150
Total protein (g/L)	55.8 (49.6–63)	55.8 (51.95–59.25)	0.744
Albumin (g/L)	29.94 ± 6.13	29.30 ± 5.57	0.493
Globulin (mg/L)	15.05 (8.9–21.65)	15.2 (7.88–21.45)	0.893
Urea (mmol/L)	5.38 (4.09–8.83)	4.2 (2.99–5.92)	0.000 *
Creatinine (μmol/L)	57 (47–67)	42 (33–54)	0.000 *
Serum cystatin C (mg/L)	1.07 (0.89–1.26)	0.79 (0.66–0.96)	0.000 *
Antithrombin III (mg/L)	61.90 ± 24.40	75.80 ± 19.93	0.053
Prothrombin time (s)	14.7 (13.7–16.5)	14.5 (13.8–15.7)	0.753
Prothrombin activity (%)	80 (63.5–92.5)	79 (70.5–88)	0.706
International normalized ratio	1.15 (1.05–1.34)	1.15 (1.08–1.25)	0.733
Activated partial thromboplastin time (s)	42.4 (36.7–47.7)	42.6 (38.3–49.75)	0.488
Plasma fibrin (g/L)	4.56 ± 1.76	4.96 ± 1.64	0.214
Fibrinogen degradation products (μg/mL)	13.03 (7.49–24.98)	17.08 (9.79–21.77)	0.383
D-dimer (μg/mL)	2.79 (1.71–6.12)	3.08 (1.45–5.2)	0.987
C-reactive protein (mg/L)	44.95 (14.93–105.75)	52.25 (13.9–133.48)	0.444
Procalcitonin (ng/mL)	0.2 (0.08–0.71)	0.2 (0.07–0.58)	0.453
B-type natriuretic peptide (pg/mL)	141 (54.25–307.25)	61 (36–219.5)	0.136

* Used to mark the result at *p*< 0.05. Continuous variables that conform to the normal distribution are represented by the mean ± standard deviation; continuous variables that do not conform to a normal distribution are represented by the median (25th–75th percentile); categorical variables are expressed by frequency.

**Table 2:** ARC and NARC clinical data after propensity score matching.

Items	ARC (N = 76)	NARC (N = 81)	*p*-Value
Gender (Male/Female)	29/22	33/18	0.190
Age (years)	59 (53–65)	56 (46–61)	0.063
Vancomycin concentration (mg/L)	14.1 (11.9–17.2)	7.3 (5.6–8.5)	0.000 *
BMI (kg/m^2^)	24.22 (22.15–27.1)	24.46 (22.86–26.3)	0.817
Creatinine clearance (ml/min)	9.27 (6.55–13.89)	8.62 (4.61–12.8)	0.238
White blood cells (10^9^/L)	1.22 (0.82–1.73)	1.2 (0.66–1.85)	0.608
Lymphocyte count (10^9^/L)	6.68 (3.89–10.33)	5.41 (1.65–8.52)	0.064
Neutrophil count (10^9^/L)	0.55 (0.4–0.84)	0.56 (0.21–0.72)	0.469
Monocytes count (10^9^/L)	75.6 (64.3–87.95)	69.6 (56–82.5)	0.086
Proportion of neutrophils (%)	3.16 (2.8–3.89)	3.42 (2.69–4.03)	0.820
Red blood cells (10^12^/L)	98.12 ± 20.56	96.37 ± 29.21	0.731
Hemoglobin (g/L)	203 (96.5–308.75)	227 (113–332.5)	0.869
Platelet count (10^9^/L)	21.5 (16.25–47.5)	26 (14–61)	0.471
Aspartate aminotransferase (U/L)	32 (18.75–57)	31.5 (20–56)	0.869
Alpine aminotransferase (U/L)	88.5 (69.5–128.75)	90.5 (61.75–129.75)	0.687
Alkaline phosphatase (U/L)	47 (26.5–97.75)	62.5 (34–98.25)	0.343
Glutamyl transpeptidase (U/L)	57.05 (49.98–63.8)	56.1 (53.9–60.53)	0.677
Total protein (g/L)	30.10 ± 6.19	29.658 ± 4.75	0.689
Albumin (g/L)	14.65 (8.6–21.75)	15.4 (8.2–21.2)	0.971
Globulin (mg/L)	6.5 (4.55–10.02)	3.97 (2.81–5.47)	0.000 *
Urea (mmol/L)	61 (50–70)	42 (34–50)	0.000 *
Creatinine (μmol/L)	1.09 (0.91–1.45)	0.82 (0.64–0.98)	0.000 *
Serum cystatin C (mg/L)	62.18 ± 15.39	42.06 ± 11.24	0.085
Antithrombin III (mg/L)	14.65 (13.85–17.48)	14.5 (13.7–15.5)	0.547
Prothrombin time(s)	79.5 (57.5–89.25)	80 (71–89)	0.558
Prothrombin activity (%)	1.16 (1.07–1.43)	1.14 (1.07–1.24)	0.566
International normalized ratio	40.85 (36.83–48.03)	42.1 (38.3–50)	0.347
Activated partial thromboplastin time (s)	4.57 ± 1.86	5.20 ± 1.48	0.119
Plasma fibrin (g/L)	13.03 (8.4–24.4)	17.22 (10.48–20.93)	0.423
Fibrinogen degradation products (μg/mL)	2.79 (1.7–6.19)	2.49 (1.38–4.58)	0.326
D-dimer (μg/mL)	53.5 (16.8–116.8)	39.4 (10.13–127.23)	0.911
C-reactive protein (mg/L)	0.2 (0.09–0.64)	0.17 (0.07–0.39)	0.194
Procalcitonin (ng/mL)	144 (58–332)	129.5 (42.25–237.25)	0.429
B-type natriuretic peptide (pg/mL)	29/22	33/18	0.190

* Used to mark the result at *p* < 0.05. Continuous variables that conform to the normal distribution are represented by the mean ± standard deviation; continuous variables that do not conform to a normal distribution are represented by the median (25th–75th percentile); categorical variables are expressed by frequency.

**Table 3:** Repeatability, Replicability, and Stability results for QC samples.

RSD (%)	837.8305 @0.85		165.0791 @3.63		563.3502 @5.24		431.3025 @7.68		1485.992 @10.74		328.1513 @13.64	
	Area	RT	Area	RT	Area	RT	Area	RT	Area	RT	Area	RT
System repeatability	2.62	0.12	0.90	0.03	2.44	0.02	1.65	0.09	2.56	0.06	2.08	0.06
Method replicability	2.55	0.70	1.90	0.30	3.37	0.14	1.56	0.07	4.50	0.03	1.71	0.02
4 h Stability	3.46	0.23	0.69	0.05	1.43	0.00	1.60	0.03	2.61	0.05	1.50	0.04
8 h Stability	4.49	0.30	4.52	0.53	1.24	0.04	2.67	0.08	1.74	0.09	1.42	0.07
12 h Stability	3.54	0.19	0.71	0.21	1.34	0.03	2.75	0.10	2.20	0.02	1.94	0.01

RSD: Relative standard deviation. The relative standard deviation of the peak area or RT of the fragment ion in the sample is divided by the corresponding mean, and the result is expressed as “%”.

**Table 4:** Differential metabolites between the ARC and NARC groups.

No.	*m*/*z*@RT	Compound Name	Molecular Formula	HMDB ID	KEGG ID	Up/Down	*p*-Value
1	199.0335@0.90	O-Phosphohomoserine	C_4_H_10_NO_6_P	HMDB0003484	C01102	Up	0.000
2	725.6688@0.93	Galactosylceramide (d18:1/18:1(9Z))	C_42_H_79_NO_8_	HMDB0010714	C02686	Down	0.000
3	284.8999@0.94	Xanthosine	C_10_H_12_N_4_O_6_	HMDB0000299	C01762	Down	0.000
4	727.664@0.96	GlcCer(d18:1/18:0)	C_42_H_81_NO_8_	HMDB0004972	C01190	Down	0.000
5	329.7503@0.97	Inosine 2′,3′-cyclic phosphate	C_10_H_11_N_4_O_7_P	HMDB0011680	C05768	Up	0.000
6	168.0808@0.98	Uric acid	C_5_H_4_N_4_O_3_	HMDB0000289	C00366	Up	0.000
7	136.0409@0.99	Hypoxanthine	C_5_H_4_N_4_O	HMDB0000157	C00262	Up	0.000
8	561.7431@1.00	Protoporphyrin IX	C_5_H_4_N_4_O	HMDB0000241	C02191	Down	0.000
9	181.0735@1.00	L-Tyrosine	C_9_H_11_NO_3_	HMDB0000158	C00082	Up	0.000
10	779.4283@1.01	PS(14:0/22:6(4Z, 7Z, 10Z, 13Z, 16Z, 19Z))	C_34_H_34_N_4_O_4_	HMDB0012340	-	Down	0.000
11	983.2857@1.09	Adrenorphin	C_44_H_69_N_15_O_9_S	HMDB0059791	C16108	Down	0.001
12	173.985@1.72	L-Arginine	C_42_H_70_NO_10_P	HMDB0000517	C00062	Up	0.001
13	129.0428@1.80	Pyroglutamic acid	C_5_H_7_NO_3_	HMDB0000267	C01879	Up	0.000
14	148.052@3.60	L-Glutamic acid	C_44_H_69_N_15_O_9_S	HMDB0000148	C00025	Down	0.000
15	242.1244@4.82	Tetrahydrobiopterin	C_6_H_14_N_4_O_2_	HMDB0000027	C00272	Down	0.000
16	258.1433@5.13	4a-Hydroxytetrahydrobiopterin	C_9_H_15_N_5_O_4_	HMDB0002281	C15522	Up	0.001
17	352.2329@6.41	Prostaglandin E2	C_25_H_40_N_2_O_6_S	HMDB0001220	C00584	Down	0.000
18	332.1375@6.48	17a-Hydroxypregnenolone	C_5_H_9_NO_4_	HMDB0000363	C05138	Up	0.000
19	779.5233@7.25	3-O-Sulfogalactosylceramide (d18:1/16:0)	C_9_H_15_N_5_O_3_	HMDB0012313	C06125	Down	0.000
20	398.2013@9.07	PC(22:5(7Z, 10Z, 13Z, 16Z, 19Z)/14:1(9Z))	C_9_H_15_N_5_O_4_	HMDB0008690	C00157	Down	0.000
21	474.282@9.36	10-Formyltetrahydrofolate	C_20_H_32_O_5_	HMDB0000972	C00234	Down	0.000
22	496.3461@9.64	Leukotriene D4	C_21_H_32_O_3_	HMDB0003080	C05951	Up	0.000
23	852.6166@9.68	Heme A	C_49_H_56_FeN_4_O_6_	HMDB0006901	C15670	Up	0.000
24	330.2389@9.88	Deoxycorticosterone	C_40_H_77_NO_11_S	HMDB0000016	C03205	Up	0.000
25	565.299@10.01	CDP-glucose	C_44_H_76_NO_8_P	HMDB0003369	C00501	Up	0.000
26	457.8033@10.14	FMNH2	C_20_H_23_N_7_O_7_	HMDB0001142	C01847	Down	0.000
27	893.6207@10.15	Octanoyl-CoA	C_25_H_40_N_2_O_6_S	HMDB0001070	C01944	Down	0.000
28	541.3042@10.19	Formamidopyrimidine nucleoside triphosphate	C_10_H_18_N_5_O_15_P_3_	HMDB0006822	C05922	Down	0.000
29	1338.8467@10.21	Ganglioside GA1 (d18:1/24:0)	C_25_H_40_N_2_O_6_S	HMDB0004909	C06136	Up	0.000
30	1142.8156@12.78	Chenodeoxycholoyl-CoA	C_45_H_74_N_7_O_19_P_3_S	HMDB0006292	C05337	Down	0.000
30	414.2053@10.45	4,4-Dimethyl-5a-cholesta-8-en-3b-ol	C_21_H_30_O_3_	HMDB0006840	C03541	Up	0.000
31	656.4657@10.83	PA(P-16:0e/18:2(9Z, 12Z))	C_15_H_25_N_3_O_16_P_2_	HMDB0011155	C15647	Up	0.000
32	907.6382@10.83	3-Oxooctanoyl-CoA	C_29_H_48_N_7_O_18_P_3_S	HMDB0003941	C05267	Down	0.000
33	464.8111@10.90	Testosterone glucuronide	C_17_H_23_N_4_O_9_P	HMDB0003193	C11134	Down	0.000
34	442.7967@11.02	Guanosine diphosphate	C_29_H_50_N_7_O_17_P_3_S	HMDB0001201	C00035	Down	0.001
35	526.8658@11.20	3-Carboxy-1-hydroxypropylthiamine diphosphate	C_10_H_18_N_5_O_15_P_3_	HMDB0006744	C05381	Down	0.000
36	643.9412@11.24	Phosphatidylinositol-3,4,5-trisphosphate	C_68_H_126_N_2_O_23_	HMDB0004249	C05981	Up	0.000
37	725.5357@11.51	PE(20:3(8Z, 11Z, 14Z)/P-16:0)	C_45_H_74_N_7_O_19_P_3_S	HMDB0009378	C00350	Down	0.000
38	1156.8068@11.62	Choloyl-CoA	C_29_H_50_O	HMDB0001374	C01794	Up	0.000
39	367.307@11.82	Prostaglandin G2	C_37_H_69_O_7_P	HMDB0003235	C05956	Up	0.000
41	740.0203@13.15	1-Diphosinositol pentakisphosphate	C_29_H_48_N_7_O_18_P_3_S	HMDB0012494	C11174	Down	0.000
42	718.0028@13.36	5-Methyltetrahydropteroyltri-L-glutamic acid	C_30_H_39_N_9_O_12_	HMDB0012177	C04489	Up	0.000
43	328.2594@13.52	Cyclic AMP	C_25_H_36_O_8_	HMDB0000058	C00575	Up	0.000
44	602.9041@13.56	Guanosine 3’,5’-bis(diphosphate)	C_10_H_15_N_5_O_11_P_2_	HMDB0059638	C01228	Up	0.000
45	647.4537@14.21	Cer(d18:1/24:1(15Z))	C_16_H_25_N_4_O_10_P_2_S	HMDB0004953	C00195	Down	0.002

**Table 5:** Basic information for the included GEO datasets.

Dataset	Disease	Platform	Experimental Group	Healthy Control Group
GSE11375	Trauma	GPL570	158	26
GSE37069	Burns	GPL570	132	37
GSE57065	Burns	GPL570	71	25
GSE19743	Sepsis	GPL570	27	28
GSE28750	Sepsis	GPL570	10	20

**Table 6:** Metabolite-metabolite species sub-information table.

ID	Name	Degree	Betweenness	Expr.
C00025	L-Glutamic Acid	20	115.4780104	−4.7288723
C00062	L-Arginine	17	99.79187188	4.249939
C00002	Adenosine Triphosphate	16	110.4030875	NA
C00006	NADP	14	62.6121986	NA
C00035	Guanosine diphosphate	13	43.00154164	−4.1962013
C00082	L-tyrosine	12	30.70040727	2.531392
C00004	NADH	12	51.46153831	NA
C00366	Uric acid	10	30.76239229	12.050512
C00272	Tetrahydrobiopterin	10	22.48090304	−9.704965
C00262	Hypoxanthine	10	20.86003946	7.3225327
C00584	Prostaglandin E2	9	46.96623783	−5.0245094
C00575	cAMP	9	34.11316313	6.905841

Degree: Degree centrality refers to the number of nodes directly connected to the node. A higher degree value indicates greater degree centrality for that point, which is more important in the network. Betweenness: Refers to the number of shortest paths through nodes in the network. Expr.: Expression quantity. Positive values indicate upregulated and negative values indicate downregulated. “NA” indicates that the metabolite is not included in the differential metabolite results of this study.

**Table 7:** Results for the joint-pathway analysis of differential metabolites and genes.

Pathway Name	Expected	Hits	*p*-Value	Impact
Nitrogen metabolism	0.1967	2	0.0154	0.4444
Porphyrin and chlorophyll metabolism	1.0426	4	0.0191	0.0962
Arachidonic acid metabolism	1.5934	5	0.0199	0.1500
Sphingolipid metabolism	1.1410	4	0.0257	0.4912
Folate biosynthesis	1.2000	4	0.0303	0.3333
Fructose and mannose metabolism	0.7869	3	0.0424	0.1539
Aminoacyl-tRNA biosynthesis	1.4557	4	0.0555	0.0548
Neomycin, kanamycin and gentamicin biosynthesis	0.0787	1	0.0764	0.6667
Glutathione metabolism	1.1016	3	0.0959	0.1818
Arginine biosynthesis	0.5312	2	0.0975	0.3462
Purine metabolism	3.2656	6	0.1036	0.1879
One carbon pool by folate	0.6098	2	0.1230	0.4333
D-Glutamine and D-glutamate metabolism	0.1967	1	0.1805	0.3333
Phenylalanine, tyrosine and tryptophan biosynthesis	0.2164	1	0.1967	0.6000
Starch and sucrose metabolism	0.8459	2	0.2066	0.1429
Glycerophospholipid metabolism	1.6918	3	0.2382	0.2118
Ubiquinone and other terpenoid-quinone biosynthesis	0.3344	1	0.2874	0.0625
Linoleic acid metabolism	0.3344	1	0.2874	0.0625
Phenylalanine metabolism	0.4131	1	0.3423	0.0500
alpha-Linolenic acid metabolism	0.4328	1	0.3553	0.0952
Phosphatidylinositol signaling system	1.4557	2	0.4311	0.0822
Fatty acid elongation	1.4754	2	0.4379	0.0946
Butanoate metabolism	0.5705	1	0.4399	0.0357
Arginine and proline metabolism	1.5344	2	0.4582	0.1169
Glycosylphosphatidylinositol (GPI)-anchor biosynthesis	0.6098	1	0.4619	0.1333
Histidine metabolism	0.6295	1	0.4727	0.0645
Selenocompound metabolism	0.6885	1	0.5036	0.0588
Ether lipid metabolism	0.7672	1	0.5421	0.0263
Primary bile acid biosynthesis	1.8098	2	0.5470	0.1099
Citrate cycle (TCA cycle)	0.8262	1	0.5690	0.0488
Fatty acid degradation	2.0066	2	0.6042	0.1089
Galactose metabolism	1.0033	1	0.6408	0.0800
Glyoxylate and dicarboxylate metabolism	1.1016	1	0.6755	0.0182
Glycolysis or gluconeogenesis	1.2000	1	0.7069	0.0667
Alanine, aspartate and glutamate metabolism	1.2000	1	0.7069	0.1167
Inositol phosphate metabolism	1.3574	1	0.7511	0.0441
Drug metabolism—other enzymes	1.3770	1	0.7561	0.0290
Cysteine and methionine metabolism	1.3967	1	0.7611	0.0286
Steroid hormone biosynthesis	3.9148	3	0.7644	0.0707
Amino sugar and nucleotide sugar metabolism	1.5541	1	0.7972	0.1282
Tyrosine metabolism	1.7311	1	0.8315	0.1264
Pyrimidine metabolism	1.9475	1	0.8657	0.0612

## Data Availability

The data of the transcriptomics presented in this study are openly available in Gene Expression Omnibus (GEO); the GEO accession numbers are GSE11357, GSE19743, GSE28750, and GSE37069. The data of the metabolomics presented in this study are available upon request from the corresponding author.
